# Clinical Features and Long-Term Follow-Up of Patients with Retinoblastoma in Turkish Children Older Than 5 Years of Age

**DOI:** 10.1155/2020/8148013

**Published:** 2020-01-20

**Authors:** Zafer Cebeci, Samuray Tuncer, Rejin Kebudi

**Affiliations:** ^1^Istanbul University, Istanbul Faculty of Medicine, Ophthalmology Department, Istanbul, Turkey; ^2^Istanbul University, Oncology Institute, Division of Pediatric Hematology-Oncology, Istanbul, Turkey

## Abstract

**Purpose:**

The diagnosis and management of retinoblastoma can be difficult in older children. This study reports the clinical features and long-term outcomes of such patients in a Turkish patient population.

**Methods:**

Medical records of 18 patients, between January 1992 and December 2017, were reviewed retrospectively.

**Results:**

Of 298 consecutive patients with retinoblastoma, 18 (6%) were at the age of 5 years and older. The median age at diagnosis was 9 years (range, 5–18 years). Misdiagnosis was noted in 8 patients (44.4%). Treatment options included enucleation in 16 patients (88.8%), adjuvant systemic chemotherapy in 3 (16.6%), intra-arterial chemotherapy in one (5.5%), and additional orbital irradiation in one (5.5%). After a median follow-up of 97 months (range, 6–252 months), all patients survived without any recurrence.

**Conclusions:**

Our series showed that 6% of patients with retinoblastoma were 5 years of age and older. Over a 15-year period, ocular salvage rate of 11.2% and survival rate of 100% were attained.

## 1. Introduction

Retinoblastoma is the most common intraocular malignancy in children [1, 2]. The diagnosis is usually made before the age of 5 and nearly less than 10% are older than 5 years of age [1–7]. Clinically, detection of a yellow-whitish retinal solid tumor with indirect ophthalmoscopy and ultrasonography helps us to confirm the diagnosis of a retinoblastoma in most of the cases. However, it can commonly lead to a diagnostic confusion in the differentiation of an intraocular mass in an older age group with coexisting vitreous hemorrhage, inflammation, or cataract that can obscure the view of the fundus [8]. In such cases, atypical signs can masquerade the tumor at initial admission, and diagnosis could be delayed for older children that can lead to extraocular extension of the disease [9].

According to the limited source of literature, retinoblastoma in older children has been reported to occur between 3.5–8.5% [4–7]. The epidemiologic data from centers in developing countries is also important, since most patients in these countries are admitted with advanced disease. We herein report the clinical features and long-term outcomes in retinoblastoma patients older than 5 years of age in a Turkish patient population.

## 2. Patients and Methods

The medical records of 298 consecutive retinoblastoma patients, treated in the Istanbul University, Istanbul Faculty of Medicine, Ophthalmology Department, Ocular Oncology Service, between January 1992 and December 2017, were reviewed. Institutional Ethics Committee approval and consent of parents were obtained for this retrospective study. The trial conformed to the tenets of the Declaration of Helsinki.

Retinoblastoma patients aged 5 years and older were included in the study. Demographic and clinical data, including gender, age at diagnosis (years), family history, symptoms, laterality, and status of the opposite eye, were evaluated. Prior referral diagnoses, pathology, and methods of treatment were also recorded. Tumor type was classified as endophytic, exophytic, mixed, or diffuse. Clinical staging of the tumor according to the International Classification of Retinoblastoma (ICRB) was used for analysis in this study.

Standard enucleation with minimal manupilation along with hydroxyapatite orbital implant was performed as described in the literature [10]. Histopathological examination of the specimen with hematoxylin-eosin staining was performed in all enucleated cases for final analysis of the tumor and its associated features. A tumor invasion into the optic nerve was categorized as “prelaminar,” “laminar,” “retrolaminar,” and “tumor at cut-end.” Choroidal involvement was documented as “none,” “focal,” or “massive” (defined as ≥3 mm in diameter) [11]. High-risk features were defined as invasion into the anterior chamber, iris, ciliary body, choroid (massive), optic nerve (retrolaminar), sclera, and extrascleral soft orbital tissue [12].

Systemic (intravenous chemotherapy), and focal (transpupillary thermotherapy, cryotherapy, external beam radiotherapy) treatment options, follow-up period (months), and the final status of the patients were recorded.

## 3. Results

Out of 298 retinoblastoma patients, 21 eyes of 18 (6%) children aged 5 years or older at initial diagnosis were included in this study. Demographic and clinical data of 18 patients are summarized in Table 1. Fifteen patients (83.3%) had unilateral and 3 patients (16.7%) had bilateral involvement. Family history was negative for all patients. The median age at diagnosis was 9 years (range, 5–18 years). Thirteen patients (72.2%) were male and 5 (27.8%) were female. The median time interval between the first symptoms to referral was 2 months (range, 1 week-17 months).

Decreased vision was the most common presenting symptom that was noted in all patients (100%), followed by leukocoria in 6 (33.3%) and strabismus in 5 (27.8%). Decreased vision was the only symptom in 9 (50%) patients.

The most common presumed diagnoses before referral to our clinic were uveitis in 2 patients (11.1%), endophthalmitis in 2 (11.1%), and Coats' disease in 1 (5.5%). In 3 patients (16.6%), no specific diagnosis was made. Only ten patients (55.5%) were referred to us with the suspicion of retinoblastoma. Before referral, two patients (11.1%) had prior vitrectomy with a presumed diagnosis of endophthalmitis.

**Figure 1:**
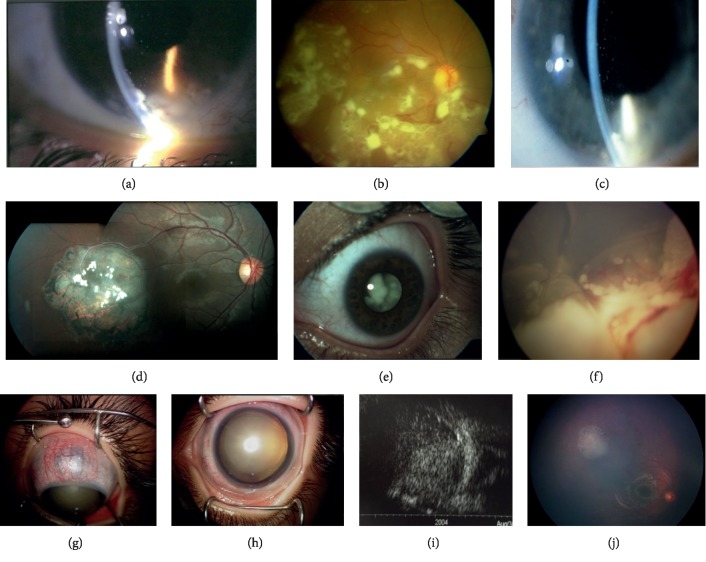
(a) Case #1. Anterior segment photo of the right eye in a 12-year-old-male patient showing multiple, round, whitish cystic lesions (pseudocysts) in the anterior chamber. (b) Fundus photo of the same patient demonstrating similar pseudocyts over the inferior half of the retinal surface. (c) Case #7. Anterior segment photo of the left eye in a 7-year-old female patient with nonconfluent pseudocysts in the anterior chamber. (d) Case #3. A 14-year-old male patient presented with a temporally located spontaneously regressed retinoblastoma in the right eye. (e) Anterior segment photo of the same patient demonstrating group E, endophytic retinoblastoma extending just behind the lens in the left eye. (f) Case #13. Fundus photo of the left eye in a 9-year-old female patient showing mixed endophytic-exophytic retinoblastoma with vitreous seeds in the inferior half of the fundus and total retinal detachment. (g–i) Case #14. A 9-year-old male patient was referred with a presumed diagnosis of Coats' disease in the left eye. Anterior segment photo showing rare coexistence of anterior staphyloma due to neovascular glaucoma (g), cataract, and ectropion uvea (h). Ultrasonography of the left eye demonstrating solid tumor with calcifications filling the vitreous cavity (i). (j) Fundus photo of the same patient showing a spontaneously regressed retinoblastoma located superotemporally in the fellow (right) eye.

Fellow eyes of two bilateral cases (9.5%) presented with spontaneously regressed retinoblastoma (Table 1, case 3 and 14). In remaining 19 eyes, there was an active intraocular tumor. Tumor growth pattern was endophytic in 6 eyes (28.6%), exophytic in 3 (14.3%), mixed endophytic-exophytic in 8 (38.1%), and diffuse infiltrating in 2 (9.5%) (Figure 1). According to the ICRB classification, 16 eyes (84.2%) presented with group E, 2 (10.5%) eyes with group D, and 1 eye (5.3%) with group B tumor. Two patients with diffuse type (Table 1, case 1 and 7) were referred to us with the diagnosis of endophthalmitis and presented with anterior chamber involvement, showing pseudohypopyon. Iris neovascularization was demonstrated in four patients (22.2%).

Enucleation was conducted as the primary surgical treatment in 16 (88.8%) patients. One group D eye managed with 2 sessions of intra-arterial chemotherapy (melfelan and topotecan) (Table 1, case 18). One group E eye could be salvaged with chemoreduction and external beam radiotherapy (Table 1, case 11). In one patient, the fellow eye harboring group B tumor was controlled with six cycles of chemoreduction and transpupillary thermotherapy (Table 1, case 10).

Histopathology of the enucleated eyes confirmed the diagnosis of retinoblastoma in all 16 enucleated eyes. Of 16 enucleated eyes, optic nerve invasion was seen in two eyes (12.5%), choroidal invasion in 5 (31.3%), and anterior chamber invasion in 2 (12.5%). Optic nerve invasion was prelaminar in one eye (Table 1, case 2) and retrolaminar in the other (Table 1, case 14). Choroidal invasion was focal in three eyes (Table 1, case 3, 6, and 12) and massive in two (Table 1, case 7 and 14). Anterior chamber invasion was detected in two eyes (Table 1, case 1 and 7). After enucleation, three patients (18.75%) received adjuvant systemic chemotherapy due to the presence of high-risk histopathological feature(s). One patient (Table 1, case 1) had additional radiotherapy due to the prior history of multiple intraocular surgeries. After a median follow-up of 97 months (range, 6–252 months), all patients (100%) survived without recurrence.

## 4. Discussion

Retinoblastoma is rare in older children, ranging from 3.5% to 8.5% of all retinoblastomas in different series [4–7]. In a recent report from China, it was shown that 47 (3.9%) of the 1,205 retinoblastoma patients were aged 5 years or older [7]. In our series over a 15-year period, out of 298 consecutive children with retinoblastoma, 6% were older than 5 years of age. Different from the previously reported literature, bilateral involvement (three patients (16.66%)) was higher in our series [4, 6, 7]. Misdiagnosis before referral was noted in 44.4% of our cases. Histopathology showed high-risk features in 3 (18.75%) of the enucleated eyes. Despite a high rate of misdiagnosis/late diagnosis and advanced stage, all patients survived with no recurrence after a median follow-up period of about 8 years.

In retinoblastoma patients older than 5 years of age, the disease has been assumed to occur as a sporadic pattern. This finding was supported by the unilateral presentation in all cases [4, 6, 7]. However, in a retrospective review by Karcioglu et al. [5], designed from the Tumor Registry of King Khaled Eye Specialist Hospital, Saudi Arabia, 4 of 18 patients (22%) presented with bilateral involvement. Similar to this study, our findings showed that 3 of 18 patients (16.6%) showed bilateral disease at initial presentation. Despite advanced disease (Group E) in the mainly affected eyes, the fellow eyes showed either spontaneously regressed retinoblastoma (in two cases) or early stage group B intraocular tumor (in one case). After 12 and 77 months of follow-up in these two cases, fellow eyes with spontaneously regressed retinoblastoma remained stable.

Before the age of 5, leukocoria and strabismus are the classical presenting signs of retinoblastoma [3, 6]. However, atypical symptoms such as decreased vision, pain, or eyelid edema generally dominate the clinical presentation in older children [5]. Shields et al. [4] reported that the initial symptoms were decreased vision (35%) and leukocoria (35%) in patients aged between 5 and 18 years. In a series of Karcioglu et al. [5], seven out of 18 children older than 5 years of age (39%) had a symptom of leukocoria, and 11 (61%) had other symptoms such as poor vision, proptosis, and pseudouveitis. Aguirre Neto et al. [6] found leukocoria (50%) as the most prevelant symptom, followed by decreased vision in 33% of the patients. In a recent study, Chang et al. [7] evaluated 47 retinoblastoma patients aged between 5 and 14 years and found that 43% of the cases complained about visual disturbances, and 13% reported pain. In our series, all patients had a complaint of decreased vision at initial admission. Only 37.5% of patients presented with leukocoria. Distinct from the series by Chang et al. [7], none of our patients reported pain in their initial examination.

In older retinoblastoma children, atypical signs such as hyphema, hypopyon (“pseudohypopyon”or cyst-like tumor aggregates), uveitis, endophthalmitis, orbital cellulitis, and vitreous hemorrhage may mimic intraocular inflammation or traumatic ocular syndrome. This can frequently cause misdiagnosis for intraocular tumors in older children [4–9]. Systemic investigations, especially in pediatric patients presenting with uveitis-like signs, and surgical intervention(s) may cause further delay for the exact diagnosis of retinoblastoma, may complicate the clinical presentation, and may lead to the progression of disease with increased risk for higher mortality [13, 14]. According to the literature, the misdiagnosis rates are reported as 17–32% in different series. Shields et al. reported a rate of 27%, Karcioglu et al. 17%, Neto et al. 31.2%, and Chang et al. 25.5% in children older than 5 years of age with retinoblastoma [4–7]. The misdiagnosis rate before referral was 44.4% and quite high in our series of older children. Moreover, two of our patients had pars plana vitrectomy with the presumed diagnosis of endophthalmitis. Both patients presented with anterior chamber pseudocysts, and one also had intravitreal pseudocysts [13]. The nonconfluent nature of the clumping cells with central necrosis (“pseudocyts”), with or without vitreous hemorrhage, should alert the clinician to a diagnosis of retinoblastoma, rather than uveitis or endophthalmitis.

The present literature refers that the absence of pain, conjunctival hyperemia, synechiae, and cataract should alert the clinician to suspect retinoblastoma rather than intraocular inflammation in older children [9]. However, in our series, a 9-year-old boy (shown in Table 1, patient no.14 and Figures 1(g)–1(j)) presented with conjunctival hyperemia, anterior scleral staphyloma due to neovascular glaucoma, ectropion uvea, and mature cataract. Before referral, he had a prior history of 15 months follow-up with the diagnosis of Coats' disease in his left eye. Despite these previously unreported atypical findings, the detection of a solid tumor with calcification under B-scan ultrasonography in the left eye and a spontaneously regressed tumor in the fellow eye led us to a diagnosis of a retinoblastoma rather than Coats' disease. The presence of neovascular glaucoma, scleral staphyloma, and cataractous changes indicates a chronic process of tumoral involvement.

The treatment of choice is enucleation in older children with advanced stage retinoblastoma [4–7]. Except for few patients, most of the reported older cases in the literature had enucleation due to the previously mentioned reasons [4–7]. Shields et al. [4] performed enucleation in 24 of 26 children (92.3%). Only two eyes (7.7%) could be saved with external beam radiotherapy. In a series of 18 patients reported by Karcioglu et al. [5], 63.6% of eyes were enucleated as primary treatment, and 36.4% were treated with other modalities. Aguirre Neto et al. [6] performed enucleation in all 15 eyes with retinoblastoma due to the advanced stage at diagnosis. In our series, our primary treatment modality was consistent with the existing literature, and 93.8% of our ICRB group E patients had enucleation. Ocular salvage could be achieved in one patient with systemic chemoreduction and further external beam radiotherapy.

There are limitations to our study including its retrospective design, absence of molecular genetic analysis, and the lack of a population-based rate that can be generalized to the whole country. The strength of our study is that it includes a series of cases with the longest follow-up compared with other studies in the literature [4–7].

In conclusion, this study reports the clinical characteristics and long-term outcomes of retinoblastoma patients older than 5 years of age from a tertiary referral center in Turkey. Our series showed that 6% of children were older than 5 years of age. Although family history was lacking, bilateral presentation was detected in about one‐sixth of our patients. The high rate of misdiagnosis in our series emphasizes the need to increase the awareness of ophthalmologists, especially in resource-limited countries, that retinoblastoma may occur in older children. Despite rarity of retinoblastoma after 5 years of age, the clinician should search for a solid tumor (with calcification) or a plaque-like thickening (without calcification) even in patients with atypical clinical presentations, including uveitis, endophthalmitis, or vitreous hemorrhage.

## Figures and Tables

**Table 1:** Demographic and clinical features of retinoblastoma (RB) in 18 children older than 5 years of age.

Case no/age (years)/gender	Laterality	ICRB group	Fellow eye status	Referral diagnosis	Time to referral (mos)	Prior treatment elsewhere	Treatment for RB	F/U (mos)	Final status
1/12/M	U	E	Nl	Endophthalmitis	17	PPV (5), PE + PCIOL, IOL removal, and iv. amphotericin B (2)	E + C + RT^ψ^	99	AWD
2/7/F	U	E	Nl	Uveitis	1	—	E^*∗*^	129	AWD
3/14/M	B	E	SR RB	RB	5	—	E^¶^	77	AWD
4/5/M	U	E	Nl	RB	2 wks	—	E	69	AWD
5/14/M	U	E	Nl	No specific diagnosis	5	—	E	184	AWD
6/10/M	U	E	Nl	RB	2 wks	—	E^¶^	198	AWD
7/7/F	U	E	Nl	Endophthalmitis	9	PPL + PPV	E + C^¶,ψ^	129	AWD
8/5/M	U	E	Nl	RB	3	—	E	105	AWD
9/5/F	U	E	Nl	No specific diagnosis	1	—	E	245	AWD
10/15/M	B	E	Group B	Uveitis	1	—	E (L), CRD + TTT (R)	19	AWD
11/8/M	U	E	Nl	RB	4	—	CRD + EBRT	203	AWD
12/5/M	U	E	Nl	RB	6	—	E^¶^	9	AWD
13/9/F	U	E	Nl	RB	1 wk	—	E	6	AWD
14/9/M	B	E	SR RB	Coats	15	—	E + C^∗,¶^	12	AWD
15/11/M	U	E	Nl	RB	1 wk	—	E	95	AWD
16/6/M	U	E	Nl	No specific diagnosis	2 wks	—	E	252	AWD
17/18/F	U	D	Nl	RB	6	—	E	6	AWD
18/7/M	U	D	NI	RB	2	IAC (2)	IAC	6	AWD

Abbreviations: AWD = alive without disease; B = bilateral; C = chemotherapy; CRD = chemoreduction; E = enucleation; F = female; IAC = intra-arterial chemotherapy; ICRB = International Classification of Retinoblastoma; iv = intravitreal; L = left eye; M = male; mm = millimeter; mos = months; Nl = normal; PE = phacoemulsification; PCIOL = posterior chamber intraocular implantation; PPL = pars plana lensectomy; PPV = pars plana vitrectomy; R = right eye; RB = retinoblastoma; RD = retinal detachment; RT = radiotherapy; SR = spontaneously regressed; U = unilateral; VA = visual acuity. The numbers in brackets indicate the total number of surgical interventions. ^*∗*^Histopathology with optic nerve involvement. ^¶^Histopathology with choroidal involvement. ^ψ^Histopathology with anterior chamber involvement.

## Data Availability

All data supporting this study are openly available from the corresponding author upon request.
